# Supplementary material for: Gymnodinium catenatum Paralytic Shellfish Toxin Production and Photobiological Responses under Marine Heat Waves
Source: Toxins (Basel). 2023 Feb 14;15(2):157. doi: 10.3390/toxins15020157 (PMC9967835; doi:10.3390/toxins15020157)

## Supplementary Materials

**Supplemental Table S1**. Results from post-hoc comparisons between treatments, sampling date and marine heatwave stage (function "emmeans"). C – control, I – marine heatwave category I, IV – marine heatwave category IV, fM – concentration in fM per cell, MF – molar fraction (%).

| Contrasts | Odds ratio/Estimate | Standard error | z-ratio | P-value |
|---|---|---|---|---|
| Cell concentration | | | | |
| C-I | 0.981 | 0.030 | -0.633 | 0.8020 |
| C-IV | 0.986 | 0.030 | -0.462 | 0.8891 |
| I-IV | 1.005 | 0.031 | 0.158 | 0.9863 |
| Beginning – Peak | 0.412 | 0.013 | -27.51 | **<.0001** |
| Beginning – Recovery | 0.466 | 0.015 | -23.19 | **<.0001** |
| Peak - Recovery | 1.132 | 0.029 | 4.89 | **<.0001** |
| Chain formation | | | | |
| C-I | 1.15 | 0.081 | 2.06 | **0.0989** |
| C-IV | 2.76 | 0.189 | 14.76 | **<.0001** |
| I-IV | 2.39 | 0.160 | 13.03 | **<.0001** |
| Beginning – Peak | 0.42 | 0.030 | -12.23 | **<.0001** |
| Beginning – Recovery | 0.52 | 0.037 | -9.11 | **<.0001** |
| Peak - Recovery | 1.26 | 0.077 | 3.76 | **0.0005** |
| Cell length | | | | |
| Beginning – Peak | 0.0021 | 0.000538 | 3.90 | **0.0003** |
| Beginning – Recovery | -0.0022 | 0.000538 | -4.17 | **<.0001** |
| Peak - Recovery | -0.0043 | 0.000538 | -8.07 | **<.0001** |
| Fv/Fm | | | | |
| Beginning – Peak | -0.079 | 0.016 | -4.96 | **<.0001** |
| Beginning – Recovery | -0.065 | 0.015 | -4.40 | **<.0001** |
| Peak - Recovery | 0.013 | 0.016 | 0.87 | 0.6609 |
| PSP toxicity | | | | |
| C-I | 6.98 | 2.4 | 2.905 | **0.0103** |
| C-IV | 5.93 | 2.4 | 2.468 | **0.0362** |
| I-IV | -1.05 | 2.4 | -0.437 | 0.9000 |
| Beginning – Peak | 4.54 | 2.4 | 1.890 | 0.1417 |
| Beginning – Recovery | 9.67 | 2.4 | 4.025 | **0.0002** |
| Peak - Recovery | 5.13 | 2.4 | 2.135 | 0.0829 |
| dcGTX3 (fM) | | | | |
| C-I | 0.69 | 0.174 | 3.97 | **0.0002** |
| C-IV | 0.63 | 0.174 | 3.66 | **0.0007** |
| I-IV | -0.05 | 0.174 | -0.30 | 0.9502 |
| Beginning – Peak | -0.10 | 0.174 | -0.60 | 0.8221 |
| Beginning – Recovery | 0.58 | 0.174 | 3.32 | **0.0026** |
| Peak - Recovery | 0.68 | 0.174 | 3.92 | **0.0003** |
| dcSTX (fM) | | | | |

| | | | | |
|---|---|---|---|---|
| C-I | 0.449 | 0.124 | 3.62 | **0.0008** |
| C-IV | 0.270 | 0.124 | 2.18 | 0.0737 |
| I-IV | -0.178 | 0.124 | -1.44 | 0.3203 |
| Beginning – Peak | 0.389 | 0.124 | 3.15 | **0.0047** |
| Beginning – Recovery | 0.668 | 0.124 | 5.40 | **<.0001** |
| Peak - Recovery | 0.279 | 0.124 | 2.26 | 0.0624 |
| dcSTX (MF) | | | | |
| Beginning – Peak | -3.04 | 0.587 | -5.18 | **<.0001** |
| Beginning – Recovery | -1.18 | 0.587 | -2.01 | 0.1095 |
| Peak - Recovery | 1.86 | 0.587 | 3.17 | **0.0044** |
| C1 (fM) | | | | |
| C-I | 2.38 | 0.982 | 2.42 | **0.0409** |
| C-IV | 1.80 | 0.982 | 1.84 | 0.1575 |
| I-IV | -0.57 | 0.982 | -0.58 | 0.8285 |
| C1 (MF) | | | | |
| Beginning – Peak | -3.50 | 1.77 | 2.16 | 0.1172 |
| Beginning – Recovery | 2.86 | 1.77 | -0.41 | 0.2380 |
| Peak - Recovery | 6.36 | 1.77 | 5.02 | **0.0009** |
| C2 (fM) | | | | |
| C-I | 2.82 | 0.95 | 2.97 | **0.0084** |
| C-IV | 2.06 | 0.95 | 2.17 | 0.0763 |
| I-IV | -0.76 | 0.95 | -0.80 | 0.7045 |
| Beginning – Peak | 3.90 | 0.95 | 4.11 | **0.0001** |
| Beginning – Recovery | 4.98 | 0.95 | 5.24 | **<.0001** |
| Peak - Recovery | 1.07 | 0.95 | 1.13 | 0.4949 |
| C2 (MF) | | | | |
| C-I | 2.28 | 1.4 | 1.63 | 0.2340 |
| C-IV | 3.54 | 1.4 | 2.52 | **0.0311** |
| I-IV | 1.26 | 1.4 | 0.90 | 0.6423 |
| Beginning – Peak | 3.55 | 1.4 | 2.54 | **0.0302** |
| Beginning – Recovery | 3.91 | 1.4 | 2.79 | **0.0146** |
| Peak - Recovery | 0.36 | 1.4 | 0.25 | 0.9653 |
| C3 (fM) | | | | |
| Beginning – Peak | 0.078 | 0.658 | -1.98 | 0.0783 |
| Beginning – Recovery | 0.021 | 0.658 | 1.62 | **0.0213** |
| Peak - Recovery | 0.500 | 0.658 | 3.60 | 0.8713 |
| C4 (fM) | | | | |
| C-I | 7.52 | 2.25 | 3.35 | **0.0023** |
| C-IV | 5.65 | 2.25 | 2.52 | **0.0319** |
| I-IV | -1.87 | 2.25 | -0.83 | 0.6818 |
| Beginning – Peak | 11.36 | 2.25 | 5.06 | **<.0001** |
| Beginning – Recovery | 13.19 | 2.25 | 5.87 | **<.0001** |
| Peak - Recovery | 1.83 | 2.25 | 0.82 | 0.6940 |
| C4 (MF) | | | | |
| Beginning – Peak | 8.26 | 2.62 | 3.15 | **0.0046** |
| Beginning – Recovery | -2.34 | 2.62 | -0.89 | 0.6447 |

| | | | | |
|---|---|---|---|---|
| Peak - Recovery | -10.60 | 2.62 | -4.04 | **0.0002** |

P-values < 0.05 are represented in bold.

**Supplemental Table S2.** MRM transitions used for PST analogues.

| **PST analog** | **Ion mode** | **MRM transitions** |
|---|---|---|
| C1, 2 | ESI− | 474 → 431; 394; 351 |
| C3, 4 | ESI− | 490 → 410; 392 |
| dcGTX2, 3 | ESI− | 351 → 333; 293 |
| GTX2, 3 | ESI− | 394 → 376; 351; 333; 300 |
| GTX1, 4 | ESI− | 410 → 367; 349 |
| GTX5 | ESI− | 378 → 360; 318; 306; 301; 122 |
| GTX6 | ESI− | 394 → 376; 351; 333; 300 |
| dcSTX | ESI+ | 257 → 239; 197; 126 |
| dcNEO | ESI+ | 273 → 255; 241; 213 |
| STX | ESI+ | 300 → 282; 266; 221; 216; 204 |
| NEO | ESI+ | 316 → 298; 273 |

**Supplemental Table S3.** LOD and LOQ obtained in the present study.

| | **nmol $L^{-1}$** | |
|---|---|---|
| **Compound** | **LOD** | **LOQ** |
| dcGTX2 | 0,329 | 1,097 |
| dcGTX3 | 0,045 | 0,149 |
| GTX5 | 0,444 | 1,479 |
| GTX2 | 0,147 | 0,491 |

| | | |
|---|---|---|
| GTX3 | 0,657 | 2,189 |
| GTX6 | 0,023 | 0,076 |
| GTX1 | 0,028 | 0,093 |
| GTX4 | 0,366 | 1,22 |
| C3 | 0,381 | 1,271 |
| C4 | 0,361 | 1,203 |
| C1 | 0,083 | 0,276 |
| C2 | 0,064 | 0,212 |
| dcSTX | 0,144 | 0,48 |
| dcNEO | 0,193 | 0,642 |
| STX | 0,003 | 0,01 |
| NEO | 0,007 | 0,022 |

**Supplemental Figure S1.** Correlation plots between a) all cell parameters and b) PSTs produced by *Gymnodinium catenatum* under control conditions and marine heatwave categories I and IV.

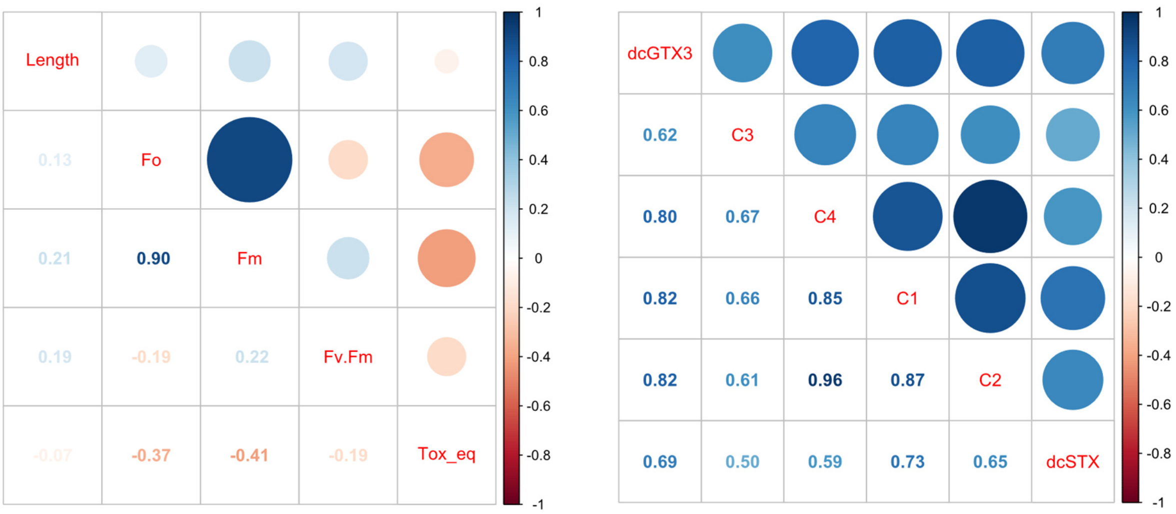

Supplement: Supplementary file 1 [file toxins-15-00157-s001.zip › toxins-2141939-supplementary.pdf]
